# Zinc Is Indispensable in Exercise-Induced Cardioprotection against Intermittent Hypoxia-Induced Left Ventricular Function Impairment in Rats

**DOI:** 10.1371/journal.pone.0168600

**Published:** 2016-12-15

**Authors:** Tsung-I Chen, Michael Yu-Chih Chen

**Affiliations:** 1 Center of Physical Education, Office of General and Basic Education, Tzu Chi University, Hualien, Taiwan; 2 Department of Cardiology, Buddhist Tzu Chi General Hospital, Hualien, Taiwan; 3 PhD Program in Institute of Medicine, Tzu Chi University, Hualien, Taiwan; Max Delbruck Centrum fur Molekulare Medizin Berlin Buch, GERMANY

## Abstract

In obstructive sleep apnea (OSA), recurrent obstruction of the upper airway leads to intermittent hypoxia (IH) during sleep, which can result in impairment of cardiac function. Although exercise can have beneficial effects against IH-induced cardiac dysfunction, the mechanism remains unclear. This study aimed to investigate the interactions of zinc and exercise on IH-triggered left ventricular dysfunction in a rat model that mimics IH in OSA patients. Nine-week-old male Sprague-Dawley rats were randomly assigned to either a control group (CON) or to a group receiving 10 weeks of exercise training (EXE). During weeks 9 and 10, half the rats in each group were subjected to IH for 8 h per day for 14 days (IHCON, IHEXE), whereas the remainder continued to breathe room air. Rats within each of the CON, IHCON, EXE, and IHEXE groups were further randomly assigned to receive intraperitoneal injections of either zinc chloride, the zinc chelator N,N,N',N'-tetrakis(2-pyridylmethyl) ethylenediamine (TPEN), or injection vehicle only. IH induced a lower left ventricular fractional shortening, reduced ejection fraction, higher myocardial levels of inflammatory factors, increased levels oxidative stress, and lower levels of antioxidative capacity, all of which were abolished by zinc treatment. IHEXE rats exhibited higher levels of cardiac function and antioxidant capacity and lower levels of inflammatory factors and oxidative stress than IHCON rats; however, IHEXE rats receiving TPEN did not exhibit these better outcomes. In conclusion, zinc is required for protecting against IH-induced LV functional impairment and likely plays a critical role in exercise-induced cardioprotection by exerting a dual antioxidant and anti-inflammatory effect.

## Introduction

The prevalence of obstructive sleep apnea (OSA) has increased over the past two decades [1]. OSA increases the risk of hypertension, stroke, heart disease, and type 2 diabetes mellitus [2]. OSA is characterized by repetitive interruption of breathing during sleep caused by recurrent upper airway collapse, resulting in intermittent hypoxia (IH) [3]. IH due to OSA can lead to cardiac dysfunction, hypertension, and heart failure [4, 5]. Development of these conditions has been attributed to increased levels of intracellular reactive oxygen species (ROS) during reoxygenation coupled [6] with reduced levels of antioxidant enzymes [7, 8]. Cardiac dysfunction in OSA is also attributed to hypoxia-associated increases in inflammation [9, 10]. High circulating levels of proinflammatory cytokines, such as tumour necrosis factor α (TNF-α), interleukin 6 (IL-6), monocyte chemoattractant protein 1 (MCP-1), and vascular cell adhesion molecule 1 (VCAM-1), have been found in OSA patients [11–14]. Moreover, higher myocardial levels of TNF-α and IL-6 have been reported in the hearts of rats subjected to IH [10].

Abnormal concentrations of zinc may exacerbate OSA-associated oxidative damage and inflammation. These results suggest that patients with OSA may benefit from zinc replacement therapy to decrease oxidative stress and reduce inflammation [9, 15]. Although zinc supplementation can provide protective antioxidant and anti-inflammatory effects [16–18], cardiac function will decline with increasing age without the stimulus of exercise [19]. Patients with mild-to-moderate OSA have been shown to benefit from regular exercise. Exercise reduces the severity of sleep apnoea [20], enhances aerobic capacity, reduces excessive daytime sleepiness [21], and improves quality of life [22]. In patients with HF and OSA, increases in left ventricular ejection fraction are associated with exercise training [23].

While exercise can improve health outcomes and quality of life in patients with OSA [24], high intensity exercise may actually worsen cardiac dysfunction. High-intensity exercise has been shown to reduce metallothionein (MT) levels, increase oxidative stress, and reduce antioxidant capacity in the hearts of rats; however, this damage was prevented by zinc treatment prior to exercise [25]. These results indicate that myocardial levels of zinc during exercise may play an important role in exercise-attenuated myocardial dysfunction induced by IH in OSA. Therefore, this study aimed to investigate the role of zinc in the cardioprotective effect of exercise in preventing left ventricular dysfunction using a rat model that mimics IH resulting from OSA.

## Materials and Methods

### Animal preparation

Sixty 9-week-old male Sprague-Dawley rats were maintained on an artificial 12-h light-dark cycle for 10 weeks, with water and food *ad libitum*. Half were randomly assigned to an exercise group (EXE) that followed a daily exercise protocol, and the remainder acted as a control group (CON). At the end of week 8, both groups were randomly divided such that half of each group underwent 2 weeks of IH, with the remainder left in room air. Thus, there were four groups: exercise combined with IH (IHEXE, N = 15); exercise with room air (EXE, N = 15); control with IH (IHCON, N = 15); and control with room air (CON, N = 15). Each group was further subdivided into three groups that received i.p. injections of vehicle (Veh; a mix of ethanol: glycerol: H_2_O in the ratio 1:3:6; n = 5) [26], zinc chloride (Zn; 10 mg∙kg^-1^; n = 5), or the membrane-permeable zinc chelator N,N,N',N'-tetrakis(2-pyridylmethyl) ethylenediamine (TPEN; 5 mg∙kg^-1^, n = 5) [18, 27]. The injections were administered to IHCON and IHEXE rats 30 min before IH exposure. The CON rats were handled in a similar manner to the other rats but did not undergo any experimental protocols. The IHEXE rats were exposed to IH during the light phase, with exercise during the dark phase on the same day. All surgical and experimental procedures were conducted following recommended procedures approved by the Institutional Animal Care and Use Committee of Tzu Chi University.

### Application of IH

The IH process has previously been described [28, 29]. In brief, rats were housed in Plexiglas cylindrical chambers (length: 28 cm; diameter: 10 cm; volume: 2.4 L) with snug-fitting lids. Using a timed solenoid valve, pure nitrogen was distributed to the chambers for 30 s to reduce the ambient inspired O_2_ fraction to 2%–6% for 2–5 s. This was followed by infusion of compressed air (for approximately 45 s), allowing the gradual return of ambient air to result in an inspired O_2_ fraction of approximate 20%. The animals were exposed to IH for 8 h per day during the dark phase for 14 consecutive days. During IH, the air flow and noise caused by the time solenoid valve and hypoxia did not change the sleep behavior of rats.

### Exercise training protocol

The exercise training protocol was modified from one used in a previous study [30, 31]. During the familiarization period, the rats initially exercised on a treadmill for 10 min∙day^-1^ at a progressively increased training intensity for 12 to 24 m∙min^-1^ with a 2% grade. At week 1, the treadmill speed was set at 24 m∙min^-1^ with a 2% grade, and the exercise time was set at 20 min∙day^-1^, which increased by 10 min∙day^-1^ until reaching 60 min∙day^-1^. Thereafter, the treadmill speed was progressively increased to 30 m∙min^-1^ for weeks 2–10. The treadmill grade was increased by 2% per week from weeks 6–10 until it reached 10% for week 9 and 10. Electrical shocks were used sparingly to motivate the animals to run. When the rat was unable to keep pace with the treadmill even after repeated application of electrical stimuli, it was removed from the treadmill and training on the next day. The rat was removed from the study if it becomes unwilling to exercise. This exercise training intensity was equivalent to approximately 70% VO_2_ max [30, 31]. For warm-up and cool-down, the rats ran on the treadmill at 15 m∙min^-1^ with a 0% grade for 5 min.

### Echocardiography for left ventricular function

Echocardiography for left ventricular (LV) function was performed under anesthesia (inhalation of 1.2%–1.5% isoflurane in oxygen). A core temperature of 37.5°C was maintained during the measurement. Transthoracic M-mode images of the LV in parasternal short-axis views were obtained at the level of the papillary muscles using a high-resolution ultrasound probe. The directly measured parameters of the LV included the end-systolic and end-diastolic dimensions (LVDs and LVDd), fractional shortening (LVFS), and ejection fraction (LVEF) [10, 32].

### Myocardial tissue preparation

Myocardial tissue preparation was as described previously [33]. Urethane has long been considered a desirable compound for providing anesthesia during physiological experiments [34]. The advantages of urethane in animal anesthesia are deep anesthesia and excellent analgesia [35] and have minimal effects on autonomic and cardiovascular systems [36]. However, urethane is not a popular anesthetic for chronic mouse preparations because it is carcinogenic and not suitable for survival surgery [37]. In this study, urethane is limited to non-survival procedures that were approved by the Institutional Animal Care and Use Committee of Tzu Chi University. Animals were euthanased with two i.p. injections of 750 mg/kg urethane (Sigma, St. Louis, MO, USA) in a 10-min interval [38]. Following a midline skin incision, the chest plate was reflected to expose the heart and lungs. The heart was lifted slightly and removed by cutting the aortic arch beyond the left subclavian artery (1.0–1.5 cm from the heart). The heart was immediately arrested and immersed in ice-cold N-2-hydroxy-ethylpiperazine-N’-2-ethanesulphonic acid (HEPES)-buffered Tyrode solution (NT), which consisted of 140 mM NaCl, 4.5 mM KCl, 1.0 mM MgCl_2_, 2.0 mM CaCl_2_, 11 mM glucose, 10 mM HEPES, and 1.2 mM KH_2_PO_4_, with pH adjusted to 7.4 at 37°C with NaOH (NT buffer). After the heart stopped beating, it was removed from the NT buffer, and the aorta was immediately affixed to a cannula attached to a syringe. The hearts were retrogradely perfused (pressure: 110 mmH_2_O) with NT buffer for 1 min to wash out blood. The LV myocardium was quickly removed, divided into six sections, snap frozen in liquid nitrogen, and stored at −80°C until use. Care was taken that the same region of the LV myocardium was harvested from all animals.

### Citrate acid synthase assay

Measurements were obtained using a commercially available Citrate Synthase Activity Colorimetric Assay Kit (BioVision, Milpitas, CA, USA), according to the manufacturer’s instructions. The absorbance of the standard and samples was read with a plate reader (Thermo Scientific, Rockford, IL, USA). All measurements were performed in duplicate on the same microtiter plate in the same setting. The total citrate acid synthase activity was normalised to the total amount of myocardium in the sample and calculated in U per milligram protein.

### Quantitative real-time polymerase chain reaction

This protocol was adapted and modified from that used in a previous study [39]. In brief, total RNA was isolated using the RNeasy Mini Kit and RNase-Free Dnase Set (Qiagen, Valencia, CA, USA) and reverse transcribed using the Omniscript RT Kit (Qiagen), as described for the previous study. The following primers were used: MCP-1 (GenBank accession number NM_031530.1), forward primer 5’-AACCAGAACCAAGTGAGATCAGA-3’ and reverse primer 5’-TGCTTGAGGTGGTTGTGGAAAA-3’; VCAM (GenBank accession number NM_012889.1), forward primer 5’-GAATGAACACTCTTACCTGTGC-3’ and reverse primer 5’-CTTGGATTCCTCTTTCCAGTTT-3’; GAPDH (GenBank accession number NM017008), forward primer 5’-TGCACCACCAACTGCTTAGC-3’ and reverse primer 5’-GCCCCACGGCCATCA-3’. PCR was performed using the SYBR Green method using TaqDNA polymerase (Invitrogen, Carlsbad, CA, USA). Fluorescence data were acquired at the end of extension. The cycle threshold value was measured and calculated using computer software (Roche LightCycler 480 II, Roche, IN, USA). We used 3 μg of total RNA to perform each reverse transcription. A 1:10 dilution of cDNA obtained in the RT reaction (25 μL total volume) was used in each quantitative real-time polymerase chain reaction (qRT-PCR). The comparative C_t_ method (2^-ΔΔCt^) was used to quantify gene expression, where ΔΔC_t_ = ΔC_t_ (sample) − ΔC_t_ (reference).

### Protein extraction and western blot analysis

This protocol was adapted and modified from our previous studies [29, 33]. Total cytosolic fraction extracts were obtained from the LV myocardium using a commercially available isolation kit (Pierce, Rockford, IL, USA), according to the manufacturer’s instructions. Total protein content was quantified in duplicate using a protein assay kit (Bio-Rad, Hercules, CA, USA) and bovine serum albumin (BSA; Sigma) standards. Samples (30 μg) were resolved on 12% SDS-PAGE for 2 h at room temperature and then electrophoretically transferred onto PVDF membranes (Amersham, Piscataway, NJ, USA). The membranes were blocked with 5% non-fat milk in Tris-buffered saline with 0.05% Tween 20 (TBS-T) at room temperature for 1 h and probed with primary antibodies for rabbit anti-TNF-α (1:1000, Abcam), IL-6 (1:1000; Chemicon, Temecula, CA, USA), metallothionein (MT, 1:1000; Santa Cruz Biotechnology Inc., Santa Cruz, CA, USA), and mouse anti-β-actin (1:5000, Chemicon) diluted in TBS-T with 2% BSA. All primary antibody incubations were conducted at 4°C overnight. The membranes were then incubated with horseradish peroxidase-conjugated secondary antibodies at room temperature for 1 h (1:5000, anti-rabbit; 1:10000, anti-mouse IgG antibody, Chemicon), and the signals were developed by enhanced chemiluminescence (Amersham ECL Prime Western Blotting Detection Reagent, GE Healthcare Life Sciences, Buckinghamshire, UK). The signals were visualised using a UVP BioSpectrum 810 (Analytik Jena AG, Jena, Germany). The resulting bands were captured by a scanner and quantified as arbitrary units (OD × band area) by ImageJ analysis software (National Institutes of Health, Bethesda, MD, USA). Protein expression in the LV myocardium was reported as the ratio of protein to β-actin.

### Measurement of myocardial oxidative stress

Oxidative stress was determined by measuring myocardial levels of lipid and protein peroxidation. Lipid peroxidation was determined by measuring myocardial levels of thiobarbituric acid reactive substances (TBARS) using a competitive enzyme immunoassay with a plate reader (Thermo Scientific Multiskan Spectrum, Rockford, IL, USA). Measurements were obtained using a commercially available TBARS kit (Cayman Chemical, Ann Arbor, MI, USA), according to the manufacturer’s instructions. Protein carbonyl content was determined by the reaction between 2,4-dinitrophenylhydrazine (DNPH) and protein carbonyls, forming a Schiff base with a plate reader. Measurements were obtained using a commercially available protein carbonyl assay kit (Cayman Chemical), according to the manufacturer’s instructions. The absorbance of the standard and samples was read with a plate reader. All measurements were performed in duplicate on the same microtiter plate with the same setting. The amount of TBARS was normalised to the total amount of myocardium in the sample and calculated in picogram∙mg^-1^ protein. The protein carbonyl content was calculated in nmol∙mg^-1^ protein.

### Measurement of myocardial antioxidant capacity

Antioxidant capacity was determined by measuring the activity levels of total superoxide dismutase (SOD), catalase, and glutathione peroxidase (GPx). Total SOD activity was determined by WST-1 formazan production using a commercially available SOD activity kit (Assay Designs, Plymouth Meeting, PA, USA). Catalase activity was determined using a formaldehyde solution as standard. Measurements were obtained using a commercially available catalase activity kit (Cayman Chemical). GPx activity was determined by the oxidation of NADPH to NAD^+^ using a commercially available GPx assay kit (Cayman Chemical). Glutathione (GSH) levels were determined from the reaction with 5,5'-dithio-bis-2-nitrobenzoic acid (DTNB). Measurements were obtained using a commercially available GSH assay kit (Cayman Chemical). The absorbance of the standard and samples was read with a plate reader (Thermo Scientific). All measurements were performed in duplicate on the same microtiter plate with the same setting, according to the manufacturer’s instructions. The total SOD, CAT, and GPx activity levels were reported in units of U∙mg^-1^ protein, and GSH levels were reported as nmol∙mg^−1^ protein.

### Statistical analyses

Statistical analyses were performed using SPSS 13.0 software (SPSS, Inc. Chicago, IL, USA). Values are presented as means and standard errors of means (SEMs). BW and echocardiographic data obtained during exercise training from week 0 to week 8 were analyzed using two-way analysis of variance (ANOVA) to assess main effects for groups (CON and EXE) and time (weeks 0–8) as the independent and repeated variables, respectively. BW and echocardiographic data during IH exposure from days 0 to 14 were analyzed using three-way ANOVA to assess main effects for groups (CON, IHCON, EXE, and IHEXE), treatments (Veh, zinc, and TPEN), and time (days 0 and 14) as the independent variables (groups and treatments) and repeated variable (time). Citrate synthase activity in the soleus, heart and ventricular weight, myocardial levels of inflammatory parameters, oxidative stress, and antioxidant capacity parameters, and myocardial levels of MT and zinc at week 10 were analyzed using two-way ANOVA to assess main effects for groups (CON, IHCON, EXE, and IHEXE) and treatments (Veh, zinc, and TPEN) as the independent variables. Tukey’s HSD protected least-significant difference test was used to determine differences between the means when comparing more than two groups. The level of significance was set at *p*<0.05 for all analyses.

## Results

### Body weight, heart weight, and echocardiographic data

**Fig 1:**
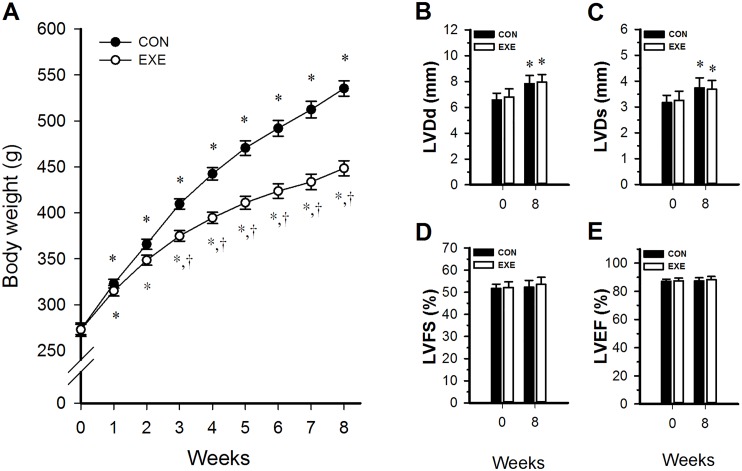
Changes during 8 weeks of exercise training in body weight (A) and in echocardiographic parameters: end-diastolic dimension of the left ventricle (LVDd) (B); end-systolic dimension of the left ventricle (LVDs) (C); left ventricular fractional shortening (LVFS) (D); and left ventricular ejection fraction (LVEF) (E). * *p*<0.05 compared with week 0. ^†^*p*<0.05 compared with the CON group. CON, controls; EXE, exercise group.

Fig 1A shows changes in body weight in the CON and EXE group from week 0 through 8. Body weight did not differ significantly between the CON and EXE groups at baseline (*p* > 0.05, Fig 1A). Between week 1 and week 8, body weights significantly increased in both groups. From week 3 to 8, body weights were significantly lower in the EXE group than in the CON group (*p* < 0.05, Fig 1A).

Fig 1B–1E show the LVDd, LVDs, LVFS, and LVEF measured at weeks 0 and 8 in the CON and EXE groups. There were no significant differences between the groups for any of these measures at week 0 (*p* > 0.05, Fig 1B to 1E). At week 8, LVDd and LVDs were significantly higher than at week 0 for both the EXE and CON groups (*p* < 0.05, Fig 1B and 1C), however, at week 8, LVDd and LVDs were not significantly different between the EXE and CON groups (*p* > 0.05, Fig 1B and 1C). Likewise LVFS and LVEF were not significantly different between the EXE and CON groups at week 8 for (*p* > 0.05, Fig 1D and 1E).

Table 1 shows echocardiographic findings on days 0 and 14 for the CON, EXE, IHCON, and IHEXE groups. On day 0, no differences in LVDd, LVDs, LVFS, and LVEF were observed across the groups injected with vehicle only (*p* > 0.05, Table 1). On day 14, LVDd was not significantly different across the four groups (*p* > 0.05, Table 1).

**Table 1:** Echocardiographic data in control and exercised rats exposed to intermittent hypoxia or room air for 14 days.

	CON			IHCON			EXE			IHEXE		
	Veh	Zinc	TPEN	Veh	Zinc	TPEN	Veh	Zinc	TPEN	Veh	Zinc	TPEN
LVDd (mm)												
Day 0	7.69±0.35	7.90±0.34	7.89±0.08	7.98±0.28	7.76±0.15	7.82±0.47	7.90±0.18	8.18±0.37	7.81±0.35	7.80±0.32	8.06±0.13	8.03±0.23
Day 14	7.74±0.28	8.23±0.17	7.77±0.39	8.03±0.15	7.21±0.33	8.08±0.25	8.26±0.29	7.66±0.19	8.23±0.25	7.99±0.19	7.88±0.52	8.70±0.22
LVDs (mm)												
Day 0	3.69±0.16	3.78±0.30	3.94±0.14	3.79±0.15	3.56±0.09	3.70±0.18	3.78±0.11	3.83±0.21	3.61±0.16	3.61±0.17	3.65±0.18	3.66±0.12
Day 14	3.49±0.20	3.72±0.05	3.66±0.18	4.60±0.11*^,^^†^	3.34±0.18^§^	4.59±0.24*^,^^¶^	3.55±0.27^‡^	3.46±0.16	3.90±0.22	3.54±0.24^‡^	3.30±0.20	4.60±0.18*^,^^§^^,^^¶^
LVFS (%)												
Day 0	51.9±1.4	52.5±1.9	50.1±1.3	52.5±1.1	54.0±1.6	52.6±1.0	52.0±1.7	53.1±1.2	53.7±1.4	53.7±1.2	54.8±1.9	54.4±1.1
Day 14	54.9±2.2	53.9±1.4	52.8±1.9	42.7±0.5*^,^^†^	53.5±2.7^§^	43.4±1.5*^,^^¶^	57.1±2.4^‡^	54.8±1.7	52.5±2.6	55.8±2.3^‡^	57.9±2.0	47.1±1.5*^,^^§^^,^^¶^
LVEF (%)												
Day 0	87.1±1.1	87.5±1.5	85.7±1.1	87.6±0.8	88.7±1.2	87.7±0.7	87.2±1.2	88.0±0.9	88.5±1.0	88.5±0.9	89.1±1.4	89.0±0.8
Day 14	89.2±1.4	88.6±0.9	87.7±1.4	79.0±0.5*^,^^†^	88.1±1.8^§^	79.5±1.5*^,^^¶^	90.5±1.4^‡^	89.2±1.2	87.3±1.8	89.7±1.5^‡^	91.1±1.3	83.0±1.3*^,^^§^^,^^¶^

Values are presented as means±SEM. CON, controls; IHCON, intermittent hypoxia; EXE, exercise; IHEXE, IH interspersed with EXE; Veh, vehicle injection; zinc, zinc chloride injection; TPEN, N,N,N',N'-tetrakis(2-pyridylmethyl) ethylenediamine injection. Echocardiographic parameters included the following: LVDd, end-diastolic dimension of the left ventricle; LVDs, end-systolic dimension of the left ventricle; LVFS, left ventricular fractional shortening; and LVEF, left ventricular ejection fraction.

*p<0.05 compared with day 0;

^†^*p*<0.05 compared with CON-Veh;

^‡^*p*<0.05 compared with IHCON-Veh;

^§^*p*<0.05 compared with vehicle injection in all four groups (CON, IHCON, EXE, or IHEXE);

^¶^*p*<0.05 compared with zinc treatment in all four groups.

On day 14, LVDs was significantly increased from day 0 values in the IHCON-Veh, IHCON-TPEN, and IHEXE-TPEN groups (*p* < 0.05, Table 1). On day 14, LVFS and LVEF in these groups had decreased significantly from day 0 values (*p* < 0.05, Table 1). On day 14, LVDs in the EXE-Veh and IHEXE-Veh groups was significantly lower than in the IHCON-Veh group (*p* < 0.05, Table 1).

In IHCON rats, LVDs in the IHCON-zinc group was significantly lower than in either the IHCON-Veh or the IHCON-TPEN groups on day 14 (*p* < 0.05, Table 1). In IHEXE rats, LVDs in the IHEXE-Veh group was significantly lower than in the IHEXE-TPEN group, and LVDs in the IHEXE-zinc group was lower than in the IHEXE-TPEN group on day 14 (*p* < 0.05, Table 1).

On day 14, LVFS and LVEF in the EXE-Veh and IHEXE-Veh groups were significantly higher than in the IHCON-Veh group (*p* < 0.05, Table 1). LVFS and LVEF in the IHCON-zinc group were significantly higher than in the IHCON-Veh group (*p* < 0.05, Table 1), and LVFS and LVEF in the IHEXE-zinc group were significantly higher than in the IHEXE-TPEN group (*p* < 0.05, Table 1). LVFS and LVEF in the IHEXE-TPEN group were significantly lower than in the IHEXE-Veh group (*p* < 0.05, Table 1). LVFS and LVEF were also significantly lower in the IHEXE-TPEN group than in the IHEXE-zinc group (*p* < 0.05, Table 1).

Table 2 shows body and cardiac tissue weights on day 0 and day 14. On day 0, EXE-Veh and IHEXE-Veh body weights were significantly lower than CON-Veh (*p* < 0.05, Table 2), and IHCON-Veh and CON-Veh body weights were not significantly different (*p* > 0.05, Table 2). With the exception of the EXE-Veh group, body weights were significantly less on day 14 than on day 0 (*p* < 0.05, Table 2).

**Table 2:** Body weight, heart weight, right ventricular weight, left ventricular weight, and citrate synthase activity in the soleus in control and exercised rats exposed to intermittent hypoxia or room air for 14 days.

	CON			IHCON			EXE			IHEXE		
	Veh	Zinc	TPEN	Veh	Zinc	TPEN	Veh	Zinc	TPEN	Veh	Zinc	TPEN
Body weight (BW, g)												
Day 0	535.2±14.4	552.2±19.4	533.4±27.0	516.0±20.3	539.4±27.9	536.0±18.1	441.0±35.2^†^^,^^‡^	446.0±11.8	456.0±14.0	431.2±9.7^†^^,^^‡^	447.0±15.9	469.4±28.5
Day 14	494.6±21.3*	500.8±18.4*	477.4±24.5*	454.8±15.0*	474.4±18.4*	454.8±13.2*	437.4±29.8	419.4±9.5*	418.6±11.7*	402.4±7.7*^,^^†^	395.4±17.5*	423.2±24.3*
Heart												
Right ventricle weight (RVW, g)	0.28±0.01	0.28±0.02	0.26±0.02	0.25±0.02	0.27±0.02	0.23±0.02	0.25±0.02	0.23±0.02	0.22±0.02	0.24±0.02	0.23±0.02	0.27±0.03
Left ventricle weight (LVW, g)	1.06±0.04	1.06±0.05	0.99±0.05	0.88±0.05^†^	0.98±0.03	0.91±0.04	0.99±0.04	0.92±0.04	0.86±0.05	0.87±0.04^†^	0.86±0.05	0.90±0.05
Heart weight (HW, g)	1.34±0.05	1.34±0.05	1.25±0.06	1.13±0.06^†^	1.26±0.04	1.14±0.05	1.24±0.05	1.15±0.06	1.08±0.06	1.11±0.05^†^	1.09±0.06	1.17±0.07
RVW/BW (mg/g)	0.57±0.01	0.56±0.02	0.56±0.02	0.57±0.02	0.58±0.02	0.52±0.02	0.57±0.02	0.56±0.04	0.55±0.04	0.61±0.04	0.59±0.04	0.67±0.04
LVW/BW (mg/g)	2.12±0.11	2.11±0.09	2.12±0.09	2.06±0.08	2.10±0.09	2.09±0.09	2.28±0.09	2.23±0.09	2.12±0.09	2.16±0.09	2.19±0.08	2.26±0.07
HW/BW (mg/g)	2.68±0.11	2.66±0.09	2.68±0.09	2.63±0.09	2.68±0.09	2.61±0.09	2.85±0.09	2.79±0.10	2.67±0.10	2.77±0.10	2.78±0.09	2.93±0.08
Soleus												
Citrate synthase activity (U/mg protein)	0.278±0.01	0.291±0.01	0.292±0.03	0.247±0.02	0.259±0.01	0.278±0.02	0.410±0.02^†^^,^^‡^	0.430±0.02	0.416±0.02	0.429±0.03^†^^,^^‡^	0.463±0.04	0.401±0.01

Values are presented as means±SEM. CON, controls; IHCON, intermittent hypoxia; EXE, exercise; IHEXE, IH interspersed with EXE; Veh, vehicle injection; zinc, zinc chloride injection; TPEN, N,N,N',N'-tetrakis(2-pyridylmethyl) ethylenediamine injection.

*p<0.05 compared with day 0;

^†^*p*<0.05 compared with CON-Veh;

^‡^*p*<0.05 compared with IHCON-Veh.

IHCON-Veh and IHEXE-Veh total heart and left ventricular (LV) weights were significantly lower than CON-Veh weights (*p* < 0.05, Table 2). Right ventricular weight, right ventricular weight/body weight, left ventricular weight/body weight, and heart weight/body weight did not significantly differ across the four vehicle-injected groups when any two were compared (*p* > 0.05, Table 2).

### Citrate synthase activity

Table 2 shows the effect of treatments on soleus muscle citrate synthase activity. EXE-Veh and IHEXE-Veh activities were significantly higher than both CON-Veh and IHCON-Veh activities (*p* < 0.05, Table 2). However, activities in the IH-Veh group did not differ significantly from the CON-Veh group (*p* > 0.05, Table 2). Furthermore, within each of the CON, IH, EXE, and IHEXE groups, activities did not significantly differ across the vehicle, zinc and TPEN subgroups when any two subgroups were compared (*p* > 0.05, Table 2).

### Inflammatory markers

**Fig 2:**
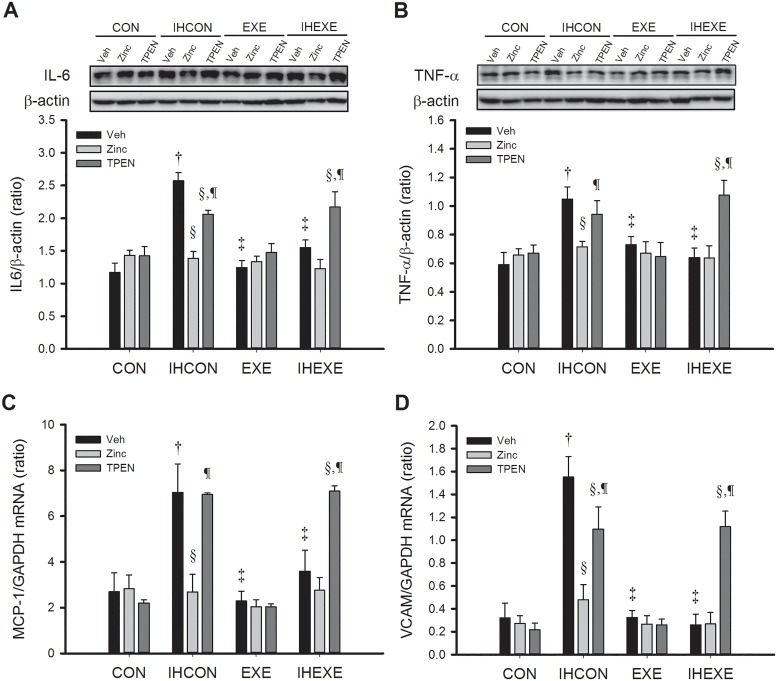
Myocardial levels of IL-6 (A) and TNF-α (B) proteins and MCP-1 (C) and VCAM (D) mRNA in control and exercised rats exposed to intermittent hypoxia or room air for 14 days. Values are mean±SEM; n = 5 per group. ^†^*p*<0.05 compared with CON-Veh; ^‡^*p*<0.05 compared with IHCON-Veh; ^§^*p*<0.05, compared with vehicle injections in all four groups (CON, IHCON, EXE, and IHEXE); ^¶^*p*<0.05 compared with zinc injections in all four groups. CON, controls; IHCON, intermittent hypoxia; EXE, exercise; IHEXE, IH interspersed with EXE; Veh, vehicle injection; zinc, zinc chloride injection; TPEN, N,N,N',N'-tetrakis(2-pyridylmethyl) ethylenediamine injection.

Fig 2 shows the effect of exercise and zinc on the expression of myocardial inflammatory markers in IH and control rats. Myocardial levels of IL-6 and TNF-α protein and MCP-1 and VCAM mRNA in the IHCON-Veh group were significantly higher than in the CON-Veh group (*p* < 0.05, Fig 2A to 2D). Levels of all four markers in the EXE-Veh and IHEXE-Veh groups were significantly lower than in the IHCON-Veh group (*p* < 0.05, Fig 2A to 2D).

Zinc and TPEN treatments had no significant effect on IL-6 and TNF-α levels in the CON and EXE groups; they were similar to the levels in the groups that received vehicle-only injections (*p* > 0.05, Fig 2A and 2B). However, zinc and TPEN treatments significantly affected levels of IL-6 and TNF-α protein and MCP-1 and VCAM mRNA in the IHCON and IHEXE groups. Levels of all four markers in the IHCON-zinc group were significantly lower than in the IHCON-Veh and IHCON-TPEN groups (*p* < 0.05, Fig 2A to 2D). IL-6 protein levels in the IHCON-TPEN group were significantly lower than in the IHCON-Veh group (*p* < 0.05, Fig 2A). The levels of all four markers in the IHEXE-TPEN group were significantly higher than in the IHEXE-Veh and IHEXE-zinc groups (*p* < 0.05, Fig 2A to 2D). In the IHEXE group, zinc injections did not significantly alter the levels of any of the inflammatory markers relative to vehicle-only injections (*p* > 0.05, Fig 2A to 2D).

### Oxidative stress

**Fig 3:**
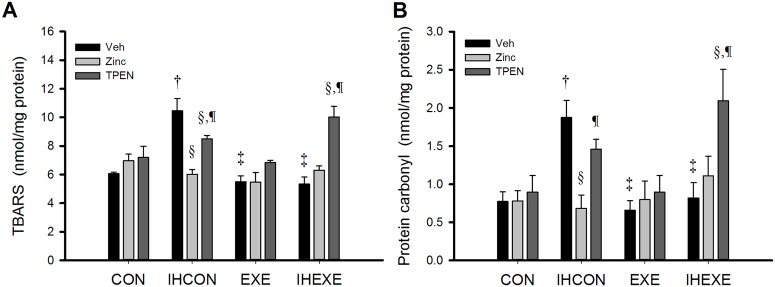
Myocardial levels of thiobarbituric acid reactive substances (TBARS): (A) as an index of lipid peroxidation and protein carbonyl; (B) as an index of protein peroxidation in control and exercised rats exposed to intermittent hypoxia or room air for 14 days. Values are mean±SEM; n = 5 per group. ^†^*p*<0.05 compared with CON-Veh; ^‡^*p*<0.05 compared with IHCON-Veh; ^§^*p*<0.05, compared with vehicle injections in all four groups (CON, IHCON, EXE, and IHEXE); ^¶^*p*<0.05 compared with zinc injections in all four groups. CON, controls; IHCON, intermittent hypoxia; EXE, exercise; IHEXE, IH interspersed with EXE; Veh, vehicle injection; zinc, zinc chloride injection; TPEN, N,N,N',N'-tetrakis(2-pyridylmethyl) ethylenediamine injection.

The effects of the various treatments on myocardial levels of TBARS and protein carbonyl are shown in Fig 3. Levels in the IHCON-Veh group were significantly higher than in the CON-Veh, EXE-Veh, and IHEXE-Veh groups (*p* < 0.05, Fig 3A and 3B). Zinc and TPEN treatments had no effect on TBARS and protein carbonyl levels in the CON and EXE groups but did have an effect in the IHCON and IHEXE groups. In the CON and EXE groups, TBARS and protein carbonyl levels in the zinc- and TPEN-treated groups were not significantly different from the vehicle-only groups (*p* > 0.05, Fig 3A and 3B). TBARS and protein carbonyl levels in the IHCON-zinc group were significantly lower than in the IHCON-Veh and IHCON-TPEN groups (*p* < 0.05, Fig 3A and 3B). Also, TBARS levels in the IHCON-TPEN group were significantly lower than in IHCON-Veh group (*p* < 0.05, Fig 3A). TBARS and protein carbonyl levels in the IHEXE-TPEN group were significantly higher than in the IHEXE-Veh and IHEXE-zinc groups (*p* < 0.05, Fig 3A and 3B). Levels in the IHEXE-zinc group were not significantly different from levels in the IHEXE-Veh group (*p* < 0.05, Fig 3A and 3B).

### Antioxidant capacity

**Fig 4:**
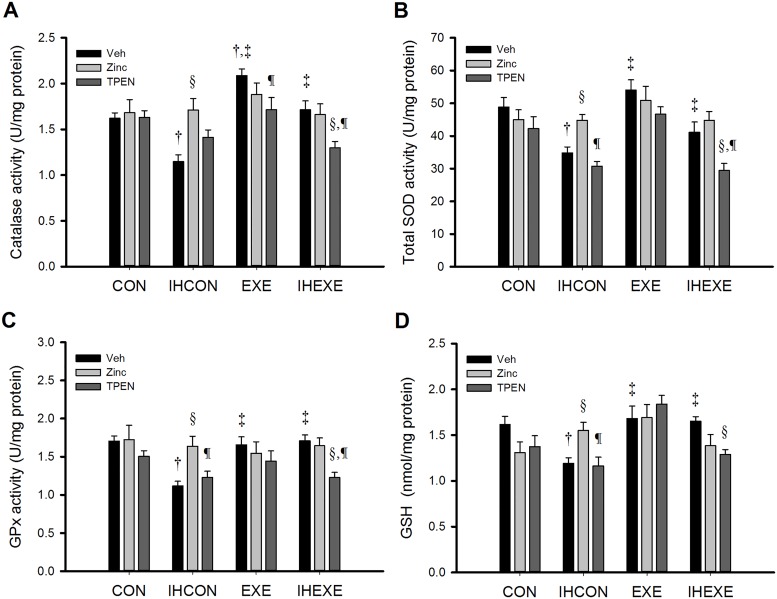
Myocardial levels of catalase (A), total superoxide dismutase (SOD) (B), glutathione peroxidase (GPx) (C), and glutathione (GSH) (D) activity in control and exercised rats exposed to intermittent hypoxia or room air for 14 days. Values are mean±SEM; n = 5 per group. ^†^*p*<0.05 compared with CON-Veh; ^‡^*p*<0.05 compared with IHCON-Veh; ^§^*p*<0.05, compared with vehicle injections in all four groups (CON, IHCON, EXE, and IHEXE); ^¶^*p*<0.05 compared with zinc injections in all four groups. CON, controls; IHCON, intermittent hypoxia; EXE, exercise; IHEXE, IH interspersed with EXE; Veh, vehicle injection; zinc, zinc chloride injection; TPEN, N,N,N',N'-tetrakis(2-pyridylmethyl) ethylenediamine injection.

Myocardial catalase, total SOD, and GPx activities and GSH levels across treatment groups are shown in Fig 4A, 4B, 4C and 4D, respectively. Levels of all four of these markers of antioxidant capacity were significantly lower in the IHCON-Veh group than in the CON-Veh group (*p* < 0.05, Fig 4A to 4D) and were higher in the EXE-Veh and IHEXE-Veh groups than in the IHCON-Veh group (*p* < 0.05, Fig 4A to 4D). In CON rats, zinc and TPEN treatment did not significantly alter levels of any marker when compared with vehicle-only treatment (*p* > 0.05, Fig 4A to 4D). In IHCON rats, the zinc group had significantly higher levels of all four markers than the vehicle-only group (*p* < 0.05, Fig 4A to 4D) and had significantly higher SOD and GPx activities and GSH levels than the TPEN group (*p* < 0.05, Fig 4B to 4D).

Catalase activities in the EXE-Veh group were significantly higher than in the CON-Veh and EXE-TPEN groups (*p* < 0.05, Fig 4A) but were not significantly different from activities in the EXE-zinc group (*p* > 0.05, Fig 4A). Total SOD and GPx activities and GSH levels in the EXE group were not significantly affected by zinc or TPEN treatment; levels in the EXE-zinc and EXE-TPEN groups were comparable to levels in the EXE-Veh group (*p* > 0.05, Fig 4B to 4D).

In IHEXE rats, catalase, total SOD, and GPx activities in the IHEXE-TPEN group were significantly lower than in the IHEXE-Veh and IHEXE-zinc groups (*p* < 0.05, Fig 4A to 4C). GSH levels in the IHEXE-TPEN group were significantly lower than in the IHEXE-Veh group (*p* < 0.05, Fig 4D). The IHEXE-zinc group did not differ significantly from the IHEXE-Veh group for any of the four markers (*p* > 0.05, Fig 4A to 4D).

### Metallothionein and zinc levels

**Fig 5:**
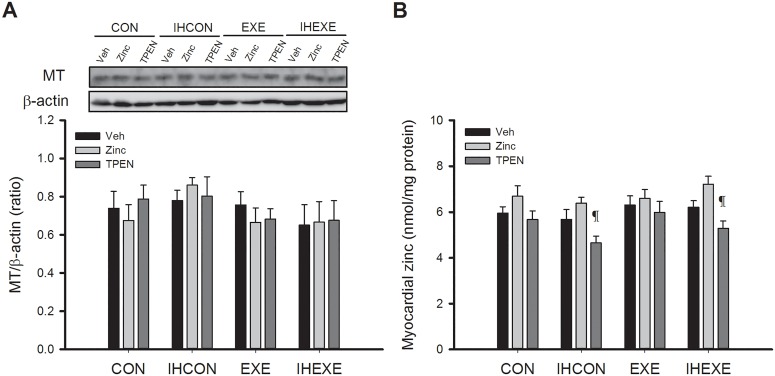
Myocardial levels of metallothionein (MT) (A) and zinc (B) in control and exercised rats exposed to intermittent hypoxia or room air for 14 days. Values are mean±SEM; n = 5 per group. ^¶^*p*<0.05 compared with zinc injections in all four groups (CON, IHCON, EXE, and IHEXE). CON, controls; IHCON, intermittent hypoxia; EXE, exercise; IHEXE, IH interspersed with EXE; Veh, vehicle injection; zinc, zinc chloride injection; TPEN, N,N,N',N'-tetrakis(2-pyridylmethyl) ethylenediamine injection.

Fig 5 shows the effects of the treatments on myocardial MT and zinc levels. MT and zinc levels were not significantly different across the four CON, IHCON, EXE, and IHEXE groups injected with vehicle (*p* > 0.05, Fig 5A and 5B). Zinc treatment did not alter MT and zinc levels relative to vehicle-only treatment in any of the four groups (*p* > 0.05, Fig 5A and 5B). TPEN treatment did not alter MT and zinc levels relative to vehicle-only treatment in the CON and EXE groups (*p* > 0.05, Fig 5A and 5B). However, TPEN treatment did significantly reduce zinc levels relative to zinc treatment in the IHCON and IHEXE groups (*p* < 0.05, Fig 5B).

## Discussion

We used a rat IH model to investigate role of zinc and exercise in preventing cardiac dysfunction resulting from oxidative stress and inflammation that results from hypoxia during OSA. Our results showed that both exercise and zinc supplementation can abolish diminished cardiac function, increased levels of inflammatory and oxidative stress markers, and attenuated antioxidant capacity resulting from IH. In rats administered the zinc-chelating agents TPEN, the protective effects of exercise were abolished.

During obstructive apneas, negative inspiratory intrathoracic pressure increases venous return, augmenting right ventricular (RV) preload, and OSA-induced hypoxic pulmonary vasoconstriction increases RV afterload. The consequent RV distension and leftward septal displacement during diastole impair LV filling [40], leading to impaired cardiac function [41]. However, IH may be a critical factor for cardiac dysfunction in animal models of OSA [5, 6]. Lower LVFS and LVEF were found in male Sprague-Dawley rats exposed to IH (4%–6% O_2_ once∙min^-1^, followed by 21% O_2_ for 8 h∙day^-1^, 5 days/week^-1^) for 5 weeks [6] and male C57BL/6 mice exposed to IH (nadir O_2_, 4%–5% once∙min^-1^, followed by 21% O_2_ for 8 h∙day^-1^, 7 days/week^-1^) for 8 weeks [32]. Short-term IH-exposures also induces impairment of LV function as reported in male Sprague-Dawley rats exposed to IH for 10 to 12 days (4%–6% O_2_ once∙min^-1^, followed by 21% O_2_ for 8 h∙day^-1^) for 10 days [5], and IH (30 s of 5% O_2_; 45 s of 21% O_2_, 6 h∙day^-1^) for 12 days [10].

ROS generated by IH can have varying impacts depending on the levels produced. Excess ROS accumulation is considered injurious. However, nonlethal levels of ROS can precondition the heart against ischaemia and reperfusion injury [42]. In isolated rat hearts, preconditioning with 10% O_2_ for 4 h reduced infarct size in isolated rat hearts [43]. Similar results were found in canine hearts subjected to 25 to 70 min of 9.5 to 10% O_2_ per day for 20 consecutive days [44, 45]. However, increases in myocardial infarct size induced by ischaemia and reperfusion were found in the hearts of rats exposed to IH with 5% O_2_ for 4 h [43] and 35 consecutive days (8 h/day) [46]. The two distinct effects of IH is probably because the increases in cellular ROS correlated with the degree of hypoxia [47]. Our findings showed that LV function was impaired by 14 days of IH exposure (2%–5% O_2_ once per 75 s, followed by 21% O_2_ for 8 h∙day^−1^).

LV performance increases with increasing exercise intensity because of an associated increase in contractility and decrease in LV afterload and preload [48]. In healthy elderly men, LV systolic function in response to afterload stress was enhanced by endurance exercise training [49]. In short-term exercise training, rats maintained higher LV-developed pressure (LVDP), and exhibited maximum rates of left ventricular pressure development (+dP/dt) and decline (–dP/dt) at all periods during ischemia and reperfusion. However, no differences were found between the control and exercise groups with regard to LVDP, +dP/dt and–dP/dt at any time during the pre-ischemia period [50]. These results indicate that exercise can prevent LV dysfunction under abnormal physiological conditions but has no effect on LV function under normal conditions. As additional examples, 3 weeks of swimming exercise (90 min, 5 days/week^−1^) in trained rats significantly lowered LVDs 24 h after myocardial infarction and also significantly attenuated LV remodelling and improved LV function. However, prior to myocardial infarction, no changes in LVDs or LV systolic function were found at the end of the exercise training [51]. Another in vivo study showed that exercise in diabetes improved LV function following 4 weeks of moderate exercise training; however, exercise did not alter cardiac parameters in control animals [52]. In hypertensive rats with prominent concentric LV hypertrophy and systolic dysfunction, combining 10 weeks of exercise training with angiotensin-converting enzyme inhibitor treatment increased LVFS by approximately 25%; however, exercise training alone did not significantly alter LV morphology or function [53]. Thus, exercise training is cardioprotective under pathological conditions [51–55] despite effects not being apparent under physiological conditions. Although the ratios of LV weight and total heart weight to body weight ratios were not altered by IH or exercise in our study, 10 weeks of exercise training resulted in lower body weights and higher soleus muscle citrate synthase activities, similar to the findings of previous studies [30, 31]. These results indicate that the exercise training protocols may have been cardioprotective against the effects of IH.

Inflammation associated with higher levels of oxidative stress may contribute to LV dysfunction [40, 56, 57]. In our study, IH raised myocardial levels of the inflammatory markers IL-6, TNF-α, MCP-1, and VCAM, similar to findings of previous studies [10, 58, 59]. Also, similar to previous studies [5, 8, 29], our study found that IH elevated levels of TBARTS and protein carbonyl, suggesting inflammation was accompanied by oxidative stress. These results suggest that IH-induced oxidative stress leads to increased myocardial inflammation.

Our results suggest that zinc plays a critical role in preventing IH-induced LV dysfunction by preventing damage due to oxidative stress and inflammation. We found that zinc administration could abolish increased oxidative stress and inflammation levels induced by IH. These results are similar to findings of a previous study that found zinc supplementation reversed diabetes-induced cardiac and renal oxidative damage, inflammation, and fibrosis [18]. In another study, cerebral ischaemia caused dramatic cytosolic zinc accumulation in ischaemic tissue that was reduced markedly by 15 mg∙kg^−1^ TPEN pretreatment [27]. In our study, IH did not cause zinc accumulation in the LV. Furthermore, IH-exposed rats administered TPEN exhibited increased levels of oxidative stress, similar to results of a previous study that reported that 5 mg∙kg^−1^ TPEN increased oxidative stress in the testicles of rats [60].

An increase in inflammatory factors has been associated with OSA [61]. IH causes a transition to increased circulating TNF-α and IL-6 levels that is a factor in the development of cardiovascular diseases in OSA patients [62, 63]. Additionally, higher plasma levels of TNF-α and IL-6 have been shown to be associated with LV dysfunction in patients with heart failure [64]. Extremely high levels of IL-1β, TNF-α, and MCP-1 induce a systemic inflammatory response and may contribute to cardiac dysfunction [65]. However, a previous study has shown that exercise could attenuate myocardial levels of TNF-α [66]. In our study, IH induced increased myocardial levels of TNF-α and IL-6 that were similar to those in carotid body inflammation and dysfunction caused by 7 days of IH in a previous study [58]. In addition, mortality from acute coronary syndromes and the risk of acute myocardial infarction are correlated with high plasma levels of MCP-1 [67]. Our study showed that myocardial levels of MCP-1 and VCAM mRNA were higher in rats exposed to IH and that this effect was abolished by zinc supplementation and exercise training. Moreover, exercise exerted an anti-inflammatory effect that was reversed by TPEN treatment. These results suggest that zinc plays a critical role in the effect of exercise in protecting against IH-induced LV inflammation.

IH can also induce the increased production of ROS and free radicals [6], which can lead to impaired cardiac function through apoptosis and necrosis of cardiomyocytes [29]. In our study, zinc treatment and exercise reduced levels of TBARS and protein carbonyl, indicators of oxidative stress, in the IH-exposed rats. Additionally, TPEN treatments in IHEXE rats abolished the positive effects of exercise on oxidative stress. Thus, our findings suggest that zinc plays a role in exercise by preventing IH-induced increases in oxidative stress.

A previous study by our group showed that IH not only increased myocardial of oxidative stress levels but also reduced antioxidant capacity [29]. Short-term exercise has been shown to increase myocardial levels of catalase activity and attenuate the myocardial oxidative damage induced by IH [33]. Additionally, in line with previous studies [33, 68], our results showed that exercise training can increase myocardial levels of catalase activity and that this effect was abolished by TPEN treatment. Thus, our findings suggest that zinc may play a role in exercise that provides antioxidant effects that prevent IH-induced reductions in myocardial antioxidant capacity.

A previous study in rats with streptozotocin-induced diabetes showed that zinc intervention can prevent the production of free radicals, the attenuation of antioxidant capacity, and muscle fatigue induced by acute exercise and diabetes [69]. However, it is still unclear whether zinc plays a role in exercise-attenuated myocardial oxidative stress and inflammation induced by OSA. Metallic zinc and MT are mutually related and interact closely, and likely protect cells from injury and promoting their rapid repair [25]. MTs are ubiquitous, small (6–8 kD), cysteine-rich, metal (Cu/Zn)-binding proteins [70, 71] with free radical scavenging properties against hydroxyl and peroxyl radicals; thus they can protect cells against cytotoxic and DNA-damaging effects [72]. Previous studies have reported that exercise induced the accumulation of MTs in normal mice spinal cords [70], rat hippocampi [73], and human muscle fibres [71]. However, a significant decrease in hepatic and cardiac MT levels was associated with 8 weeks of exercise training in spontaneously hypertensive rats and Wistar Kyoto rats [67]. Interestingly, a significant increase in MT levels was observed in the aortas of exercised SHRs in comparison with nonexercised SHRs [72]. Notably, intense exercise has been shown to attenuate the myocardial level of MT, increase the level of lipid peroxidation, reduce the activity of antioxidant enzymes, and cause calcium overload in rat hearts, leading to cardiomyocyte death; this negative outcome was prevented by zinc administration prior to the exercise [25].

Unlike in these studies, in our study myocardial levels of MT and zinc were not altered by IH and exercise. This may be due to differences in the type, duration, intensity, and period of exercise training, or from differences in the rat strain or organs. Also, compared with ischaemia and reperfusion, IH generates only mild myocardial oxidative stress in rats [33]. Additionally, the exercise intensity in our study was not high enough to generate large amounts of ROS in rat hearts. Although the zinc intervention did not increase myocardial levels of zinc in our study, TPEN treatment did result in significantly lower myocardial levels of zinc compared with rats that received zinc in the IHCON and IHEXE groups. These results indicated that low myocardial levels of zinc exacerbated IH-induced cardiac dysfunction and abolished the cardioprotective effects of exercise.

IH is believed to contribute to the pathogenesis of hypertension in OSA through mechanisms that include activation of the renin-angiotensin system [74]. The renin-angiotensin system may participate in the overproduction of reactive oxygen species associated with IH by upregulation of the actions of angiotensin II [75]. Whether the ROS-scavenging effects of zinc and exercise might be acting through this secondary mechanism was not investigated in this study. A second limitation is that the optimal levels of zinc for preventing IH-induced LV dysfunction were not explored. In this study, we found that 10 mg/kg/day zinc prevented IH-induced left ventricular dysfunction in rats. The recommended daily dosage of zinc for healthy individuals is 0.5 to 1 mg/kg/day in children and 15 to 30 mg/day in adults [76]. Whether these doses are sufficient for preventing cardiac injury in patients with OSA during high-intensity exercise needs further investigation.

## Conclusion

In this study, we used a rat model to investigate the role of zinc and exercise in preventing cardiac dysfunction due to IH in OSA. IH increased myocardial levels of inflammation and oxidative stress, which contributed to LV function impairment, and insufficient zinc levels in the myocardium exacerbated LV dysfunction. Zinc administration and chronic exercise training exerted similar cardioprotective effects against IH-induced cardiac dysfunction. Although exercise did not increase LV function under physiological conditions, under conditions of IH, exercise training attenuated cardiac damage. These cardioprotective effects of exercise were prevented by the administration of the zinc-chelator TPEN. In conclusion, zinc is required for protecting against IH-induced LV functional impairment and likely plays a critical role in exercise-induced cardioprotection by exerting a dual antioxidant and anti-inflammatory effect.
